# Fat embolism syndrome caused by fracture or liposuction: a retrospective case series of nine patients

**DOI:** 10.1080/07853890.2024.2447427

**Published:** 2024-12-30

**Authors:** Xuexin Yan, Siyao Wu, Wen Zeng, Jinliang Kong

**Affiliations:** Department of Pulmonary and Critical Care Medicine, The First Affiliated Hospital of Guangxi Medical University, Nanning, PR China

**Keywords:** Fat embolism syndrome, fracture, liposuction

## Abstract

**Background:**

Fat embolism syndrome (FES) is a rare and potentially fatal complication commonly observed after liposuction or fractures. Patients with FES often have an acute onset and a rapid course of disease. However, there is a paucity of research summarizing the clinical features of FES caused by simultaneous fracture or liposuction.

**Methods:**

A comprehensive analysis was conducted to enhance understanding of patients with FES associated with either fractures or liposuction procedures.

**Results:**

Nine patients who were diagnosed with FES were included in our study, of whom two were male. Six patients underwent liposuction, while three sustained multiple orthopaedic fractures. Postoperative liposuction complications occurred rapidly (average 1.8 h) after surgery, whereas patients with fractures experienced symptoms approximately 24 h after injury. All patients presented with respiratory insufficiency, six patients had cerebral involvement, and four patients had petechial haemorrhage. Laboratory tests revealed that six patients had leucocytosis, five patients had elevated neutrophil counts and eight patients had increased D-dimer concentrations. Seven patients exhibited decreased partial pressure of oxygen (7/9), six presented with decreased haemoglobin (6/9) and four had thrombocytopenia (4/9). Chest computed tomography (CT) revealed pneumonia with symmetrically diffuse ground glass opacities and patchy exudates in both lungs, which are the most common radiographic findings (8/9). Brain CT of five patients revealed multiple cerebral infarctions, and CT angiograms of the head and neck demonstrated corresponding vessel occlusions in one patient. All patients received supportive care, including six who received noninvasive ventilation and three who received mechanical ventilation. Additionally, seven patients were treated with corticosteroids. Eight patients survived, while one died of sustained cerebral embolism and ischaemia during treatment.

**Conclusions:**

A patient’s medical history is critical for the diagnosis of FES. Early diagnosis and timely treatment can reduce mortality, while supportive care is important, and corticosteroid therapy may be effective for early treatment.

## Introduction

Fat embolism syndrome (FES) is a rare potentially fatal complication that can develop after liposuction or after sustaining an orthopaedic fracture [1,2]. One pathophysiologic mechanism for the clinical manifestations of FES is the mechanical theory. The release of fat globules into circulation results in mechanical embolization, resulting in the occlusion of blood vessels in vital organs and subsequent compromised blood flow, ultimately leading to organ infarction and functional impairment, which may give rise to extensive cerebral or pulmonary infarctions [3]. In addition to the inherent risks associated with liposuction and fat grafting procedures involving the injection of adipose tissue into veins, a related study has shown that liposuction itself can induce adipose mobilization, thereby increasing the risk of patients developing FES [4]. Another pathophysiologic mechanism of FES is the biochemical theory. Fat globules undergo enzymatic hydrolysis, resulting in the formation of glycerol and free fatty acids, which have potential biochemical toxicity. This leads to inflammatory and immune responses that cause vasogenic oedema and haemorrhage, ultimately damaging multiple organs [5, 6]. The onset of FES occurs within 12–72 h following liposuction, while symptoms manifest between 24 and 72 h after fracture [1,7]. The clinical manifestations of this disease commonly involve the respiratory, neurological, cardiovascular and dermatological systems, which are also common in other diseases. The overall mortality rate is approximately 6–10%, often due to a delay in diagnosis caused by nonspecific symptoms [8]. Early clinical suspicion and accurate description of clinical manifestations are crucial for recognition. As most current studies are case reports, we analysed the clinical and imaging data from nine patients with FES to explore their clinical characteristics and improve understanding.

## Materials and methods

### Study population

**Figure 1.**
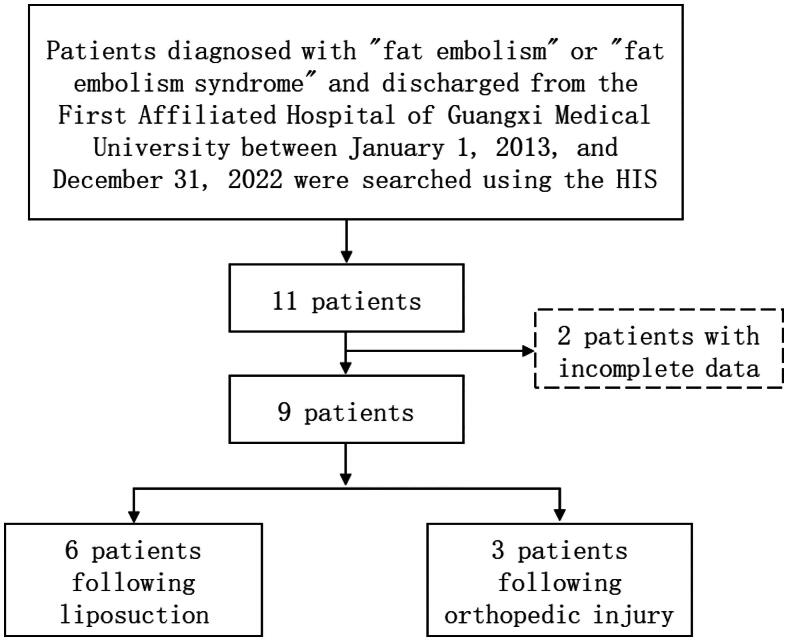
Flow diagram showing the enrolment of FES inpatients.

The systematic search was conducted at the First Affiliated Hospital of Guangxi Medical University between 1 January 2013 and 31 December 2022, utilizing terms such as ‘fat embolism syndrome’ and ‘fat embolism’. Utilizing our hospital’s unified electronic medical record system, the Hospital Information System (HIS), a total of 11 patients were identified, out of which two were excluded due to incomplete data. Consequently, a cohort comprising nine patients diagnosed with Fat Embolism Syndrome at the First Affiliated Hospital of Guangxi Medical University between 1 January 2013 and 31 December 2022 was included (Figure 1). The relevant information, including demographic data, clinical characteristics, laboratory findings, imaging manifestations, treatment and clinical outcomes, was extracted from HIS. All research procedures were performed in accordance with the Declaration of Helsinki.

### Diagnostic criteria for FES

FES is the clinical manifestation of fat embolism, which classically presents with respiratory, neurological and dermatological features. The diagnosis of FES relies on the identification of known causal triggers, recognition of clinical manifestations, interpretation of laboratory examination findings and radiological features, and exclusion of other critical illnesses [4]. The widely used diagnostic criteria consisting of clinical features and laboratory tests were proposed by Gurd and Wilson (Table 1) [9].

**Table 1.** Gurd and Wilson criteria for FES.

Fat embolism syndrome (FES = 1 major + 4 minor)	
Major criteria	Minor criteria
Hypoxia (<60 mmHg O_2_)	Pyrexia (>39 °C)
Confusion	Tachycardia (>120 beats per minute)
Petechial rash	Retinal changes (petechiae)
	Anuria or oliguria
	Anaemia (haemoglobin drop 20%)
	Thrombocytopenia (drop 50%)
	High ESR (>71 mm per hour)
	Fat macroglobulinaemia

mmHg: millimetre of mercury; O_2_: oxygen; ESR: erythrocyte sedimentation rate.

### Statistical analysis

The data are expressed as the median (interquartile range), owing to its skewed distribution. Categorical variables are expressed as percentages. We used SPSS (version 25.0) (SPSS Inc., Chicago, IL) for statistical analysis and to prepare the graphs.

## Results

### General characteristics

Nine patients diagnosed with FES, including two males with a median age of 39 years (range 24–64 years), were included in this retrospective study. Two patients had complicated hepatitis B infection. Six patients underwent liposuction, and five of whom underwent surgery were combined with other procedures, namely, breast surgery in two patients and facial fat grafting in three patients. The remaining three patients suffered orthopaedic fractures, including one closed fracture of the tibia and fibula, one fracture of the left femur and pubis and multiple fractures in another patient. The conditions of the patients who had undergone liposuction while they were still recovering from the surgery were identified (4/6, 66.7%). Patients who underwent liposuction developed conditions within a short period after surgery (average 1.8 h), while patients with fractures exhibited symptoms approximately 24 h after the initial trauma. In addition, patient 6 underwent manual reduction and external fixation 10 h after the trauma; however, he remained unconscious 24 h postsurgery. Patients 3 and 4 developed symptoms of FES during postanaesthesia recovery, while patient 9 experienced FES intraoperatively. Patient 1 manifested symptoms 1 h after surgery, patient 2 exhibited symptoms after 7 h and patient 5 exhibited symptoms after 3 h (Table 2).

**Table 2.** Demographic and clinical characteristics of nine patients with fat embolism syndrome.

Patient	Age/sex	Comorbidities	Surgery or injury	Time to first symptom after surgery (h)	Clinical syndrome	Respiratory supportive	Require ICU stay	Glucocorticoid therapy/day	Result
P1	38/F	Hepatitis B	Liposuction and breast augmentation	1	1. Respiratory insufficiency 2. Cardiovascular symptoms (tachycardia, hypotension) 3. Hematologic system (anaemia, thrombocytopenia) 4. Fever	NPPV	Yes	Methylprednisolone/1 day	Improved
P2	40/F	No	Liposuction and breast augmentation	7	1. Respiratory insufficiency 2. Cardiovascular symptoms (tachycardia, hypotension) 3. Hematologic system (anaemia, thrombocytopenia) 4. Petechial rash	NPPV	Yes	Methylprednisolone/5 day	Improved
P3	30/F	No	Liposuction and facial fat grafting	Recovering from anaesthesia	1. Respiratory insufficiency 2. Cerebral involvement 3. Cardiovascular symptoms (tachycardia, hypotension) 4. Petechial rash 5. Fever	IPPV	Yes	Methylprednisolone/2 day	Death
P4	24/F	No	Liposuction	Recovering from anaesthesia	1. Respiratory insufficiency 2. Cerebral involvement 3. Cardiovascular symptoms (tachycardia, hypotension) 4. Digestive system (nausea, vomiting, aminotransferase elevation)	NPPV	Yes	No	Improved
P5	42/F	Depressive disorder	Liposuction and facial fat grafting	3	1. Respiratory insufficiency 2. Cardiovascular symptoms (tachycardia) 3. Petechial rash	NPPV	Yes	Methylprednisolone/7 day	Improved
P6	27/M	No	Liposuction and facial fat grafting	In operation	1. Respiratory insufficiency 2. Cerebral involvement 3. Hematologic system (anaemia)	IPPV	Yes	No	Improved
P7	64/F	No	Orthopaedic injury	24	1. Respiratory insufficiency 2. Cerebral involvement 3. Ophthalmoconjunctival haemorrhage 4. Hematologic system (anaemia, thrombocytopenia)	NPPV	Yes	Dexamethasone/12 day	Improved
P8	35/M	No	Orthopaedic injury	24	1. Respiratory insufficiency 2. Cardiovascular symptoms (tachycardia) 3. Fever 4. Hematologic system (anaemia, thrombocytopenia) 5. Digestive system (aminotransferase elevation)	NPPV	Yes	Dexamethasone/2 day	Improved
P9	40/M	Hepatitis B	Orthopaedic injury	24	1. Respiratory insufficiency 2. Cerebral involvement 3. Fever 4. Digestive system (jaundice) 4. Hematologic system (anaemia)	IPPV	Yes	Methylprednisolone/10 d	Improved

P: patient; F: female; M: male; ICU: intensive care unit; NPPV: nasal intermittent positive pressure ventilation; IPPV: intermittent positive-pressure ventilation; h: hour.

### Clinical manifestations and laboratory examination findings

All nine patients presented with respiratory insufficiency, five patients had nervous system involvement and four had petechial haemorrhage. The most common clinical features were respiratory symptoms, with dyspnoea observed in seven patients, coughing with expectoration in four patients, haemoptysis in one patient and chest pain in another. Neurological symptoms and cardiovascular manifestations both accounted for 66.7% of patients. Among the patients who presented with neurological symptoms, five exhibited disturbance of consciousness, one had lalopathy and two complained of headache and dizziness. Among patients with cardiovascular manifestations, six presented with tachycardia and four presented with hypotension. All clinical features are listed in Table 3.

**Table 3.** Clinical manifestations in nine patients with fat embolism syndrome.

Characteristics	Liposuction, *N* (%)	Orthopaedic injury, *N* (%)	Abnormal patients, *N* (%)
*Clinical manifestations*			
Respiratory symptoms	6 (100)	3 (100)	9 (100)
Cough and expectoration	3 (50)	1 (33.3)	4 (44.4)
Haemoptysis	1 (16.7)	0	1 (11.1)
Stethalgia	1 (16.7)	0	1 (11.1)
Dyspnoea	6 (100)	1 (33.3)	7 (77.8)
Neurologic symptoms	3 (50)	3 (100)	6 (66.7)
Disturbance of consciousness	2 (33.3)	3 (100)	5 (55.6)
Lalopathy	0	1 (33.3)	1 (11.1)
Headache and dizziness	2 (33.3)	0	2 (22.2)
Cardiovascular symptoms	5 (83.3)	1 (33.3)	6 (66.7)
Tachycardia	5 (83.3)	1 (33.3)	6 (66.7)
Hypotension	4 (66.7)	0	4 (44.4)
Petechial haemorrhage	3 (50)	1 (33.3)	4 (44.4)
Other symptoms			
Fever	2 (33.3)	2 (66.7)	4 (44.4)
Nausea and vomiting	1 (16.7)	0	1 (11.1)
*Treatment*			
Require ICU stay(day)	6 (100)	3 (100)	9 (100)
Respiratory supportive treatment	6 (100)	3 (100)	9 (100)
Invasive mechanical ventilation	2 (33.3)	1 (33.3)	3 (33.3)
Non-invasive mechanical ventilation	4 (66.7)	2 (66.7)	6 (66.6)
Glucocorticoid therapy	4 (66.7)	3 (100)	7 (77.8)
*Result*			
Improved	5 (83.3)	3 (100)	8 (88.9)
Death	1 (16.7)	0	1 (11.1)

*N*: number; ICU: intensive care unit.

Various laboratory tests were conducted to assess the patient’s condition, including the detection of inflammatory markers, kidney function markers, liver function markers, coagulation function markers and other blood indexes due to the development of FES. The laboratory examinations revealed that seven patients exhibited hypoxemia, and the median arterial partial pressure of oxygen was 54.7 (48.4, 69.4)** **mmHg. Six patients (77.8%) exhibited leucocytosis, and five (55.6%) had neutrophilia, with median values of 12.8 (9.5, 21.4) × 10^9^/L and 10.9 (8.1, 19.3) × 10^9^/L, respectively. Six patients (66.7%) presented with decreased haemoglobin concentrations, with a median value of 98 (83.4, 124.9) g/L. Four patients had decreased platelet counts, with a median value of 121.4 (78.9, 193.0) × 10^9^/L. Elevated D-dimer concentrations were found in eight patients, with a median value of 1853 (733.5, 4549.0) ng/mL. Additionally, three patients exhibited abnormal liver function, including two with increased aminotransferase levels and one with increased bilirubin levels. All patients had normal kidney and coagulation function. The detailed data are comprehensively presented in Table 4.

**Table 4.** Laboratory examination of nine patients with fat embolism syndrome.

Variable	P1	P2	P3	P4	P5	P6	P7	P8	P9	Median (25th‒75th percentile)
PaO_2_ (mmHg)	56.7	46.4	60.3	78.5	46.8	49.9	53.5	54.7	98	54.7 (49.9, 60.3)
WBC (×10^9^/L)	23.5	11.0	13.2	26.7	12.8	9.7	6.6	9.3	19.2	12.8 (9.7, 19.2)
Neu (×10^9^ cells/L)	23.1	10.0	12.8	24.0	10.9	8.3	5.8	7.9	15.4	10.9 (8.3, 15.4)
Lym (×10^9^ cells/L)	0.2	0.4	0.3	1.5	1.3	0.9	0.4	0.5	1.2	0.5 (0.4, 1.2)
Hb (g/L)	98.1	90.9	129.2	124.6	125.1	98	80	68	86.9	98 (86.9–124.6)
PLT (×10^9^/L)	78.8	89.6	237.6	192.1	193.8	131	79	59	121.4	121.4 (79–192.1)
ALB (g/L)	43.5	33.3	40.6	40.6	29.3	28.9	35.3	31.8	44.9	35.3 (31.8–40.6)
AST (U/L)	23	30	31	175	55	40	79	171	29	40 (30–79)
ALT (U/L)	14	17	25	158	46	39	56	141	42	42 (25–56)
TBiL (mmol/L)	19	3.9	5.1	9.1	10	10.5	10	16.5	57.1	10 (9.1–16.5)
DBiL (mmol/L)	5	1.4	1.8	1.8	3.8	5.4	2.8	3.3	27.1	3.3 (1.8–5)
Cr (μmol/L)	40	76	55	85	54	67	60	70	61	61 (55–70)
PT (S)	12.7	12.4	11.6	13.6	13.0	13	11.4	11.2	12.6	12.6 (11.6–13)
APTT (S)	30.7	28.7	29.6	27.0	24.0	17	25.6	NR	25.6	26.3 (25.2–28.93)
D-dimer (ng/mL)	543	358	2451	7390	924	1420	5223	3875	1853	1853 (924–3875)
ESR (mm/H)	8	89	46	35	14	45	76	56	132	46 (35–76)

Normal range: WBC: 3.5–9.5 × 10^9^/L; Neu: 1.8–6.3 × 10^9^ cells/L; Lym: 1.1–3.2 × 10^9^ cells/L; Hb: 115–150 g/L; PLT: 125–350 × 10^9^/L; ALB: 40–55 g/L; AST: 13–40 U/L; ALT: 7–45 U/L; TBiL: 3.4–20.5 mmol/L; DBiL: 0–6.8 mmol/L; Cr: 45–84 μmol/L; PT: 9–15 s; APTT: 23–40 s; D-dimer: 0–450 ng/mL; ESR: 0–20 mm/H; P: patient; WBC: white blood cell count; Neu: neutrophil; Lym: lymphocyte count; Hb: haemoglobin; PLT: platelet count; ALB: albumin; AST: aspartic transaminase; ALT: alanine aminotransferase; TBiL: total bilirubin; DBIL: direct bilirubin; Cr: creatinine; PT: prothrombin time; APTT: activated partial thromboplastin time; ESR: erythrocyte sedimentation rate.

### Radiology features

**Figure 2.**
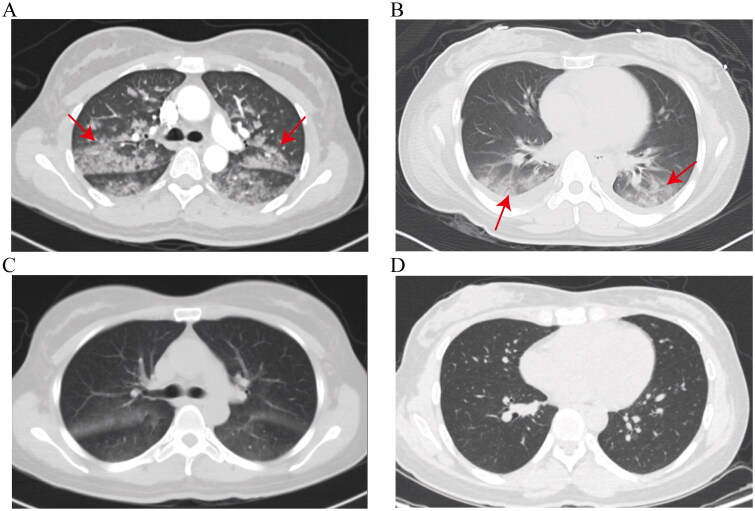
(A, C) Lung CT images of patient 1 after liposuction-induced FES. (B, D) Lung CT images of patient 4 after liposuction-induced FES. Arrows indicate the corresponding lesions. (A) Initial lung CT revealed bilateral patchy exudation and ground-glass opacities with increased density and indistinct margins in the upper and lower lobes, without pleural effusion. (B) Initial lung CT revealed symmetrical patchy and strip-like exudation with increased density and indistinct margins in both lower lobes, as well as pleural effusion. (C) The second lung CT scan, conducted two days after treatment, showed a greater degree of absorption in the lesion than in its previous state. (D) The second lung CT scan, conducted 2 weeks after treatment, showed basic absorption of the lesion.

Chest computed tomography (CT) was performed for all patients. The most common imaging manifestation was patchy exudation, which was present in all patients. Among them, five patients (55.6%) presented with symmetrical ground-glass opacities and patchy exudation bilaterally in all lobes. For instance, the initial chest CT of patient 1 revealed bilateral patchy exudation and ground-glass opacities with increased density and indistinct margins in the upper and lower lobes (Figure 2(A)). Three patients (33.4%) had similar findings limited to the bilateral lower lobes. For example, patient 4’s initial chest CT scan revealed symmetrical patchy and strip-like exudation with increased density and indistinct margins in both lower lobes, as well as pleural effusion (Figure 2(B)). One patient exhibited exudative changes solely in the right lower lobe. Furthermore, pleural effusion was detected in five patients.

Five patients underwent chest CT re-examination within 48–72 h. Four of them showed significant absorption of lung lesions, similar to the chest CT re-examination of patient 1, who showed a greater degree of absorption (Figure 2(C)), while one patient exhibited no notable changes. The remaining two patients demonstrated improved absorption upon chest CT re-examination after two weeks. For instance, the chest CT re-examination of patient 4 conducted two weeks after treatment showed basic absorption of the lesion (Figure 2(D)).

**Figure 3.**
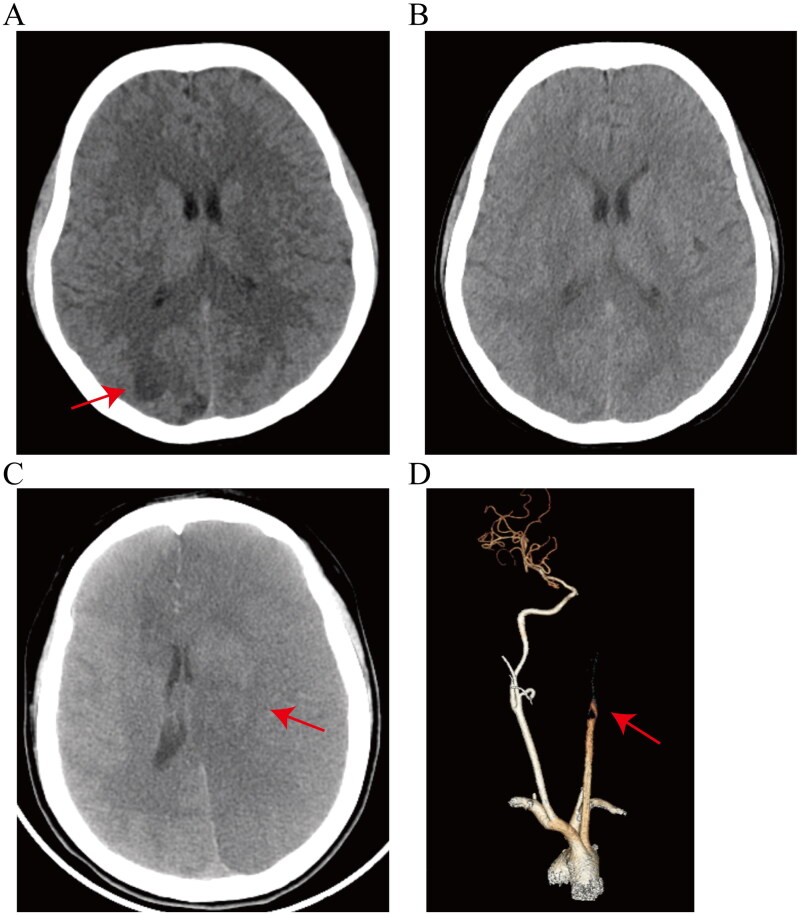
(A, B) Brain CT images of patient 4 after liposuction-induced FES. (A) Initial brain CT image showing patchy low-density shadows in the right parieto-occipital lobe, which was indicative of infarction. Arrows indicate the corresponding lesions. (B) A second brain CT scan, conducted 2 weeks after treatment, revealed basic absorption of the lesion. (C, D) Brain CT and CT angiograms of the head and neck of patient 3 after liposuction-induced FES. (C) Brain CT revealed mild swelling in the left temporal and parietal lobes, shallow sulci and fissures, blurred cortical medullary junctions, compression of the left ventriculus cerebri, and a shift of the central structures towards the right. Arrows indicate the corresponding lesions. (D) CT angiograms of the head and neck showing occlusion of the distal left common carotid artery and left internal carotid artery.

Five patients underwent brain CT scans, and the main manifestations were patchy low-density shadows with borderline ambiguity, oedema and compression of brain tissue, which were indicative of cerebral infarction. The initial brain CT scan of patient 4 revealed patchy low-density shadows in the right parieto-occipital lobe (Figure 3(A)). After a two-week period of respiratory support therapy, circulatory support therapy and cranial pressure reduction therapy, patient 4 underwent re-examination via craniocerebral CT scan which revealed significant resolution of brain lesions. Additionally, significant absorption of brain lesions was observed after a two-week therapy (Figure 3(B)). A brain CT scan of patient 3 revealed infarction in the right parieto-occipital lobe, left cerebellar hemisphere and left occipital lobe (Figure 3(C)). Moreover, CT angiograms of the head and neck demonstrated occlusion of the distal left common carotid artery and left internal carotid artery corresponding to the brain lesions (Figure 3(D)).

### Treatment and outcomes

Nine patients received respiratory support, six of whom received non-invasive ventilation and three of whom required mechanical ventilation. Patient 5 was transferred to the intensive care unit (ICU) due to respiratory failure, while patients 1, 2 and 4 were admitted to the ICU for combined respiratory and circulatory instability. Additionally, patients 3, 6, 7, 8 and 9 were transferred to the ICU because of respiratory failure accompanied by coma. Corticosteroids were administered in seven patients (77.8%) after admission to the ICU. Among these patients, five received methylprednisolone and two received dexamethasone. The dosage of methylprednisolone ranged from 1 to 1.5 mg/kg/day, and the duration of use varied from 1 to 12 days. Two patients received high doses of corticosteroids, such as 240 mg of methylprednisolone or an equally potent alternative such as dexamethasone. However, two patients experienced gastrointestinal bleeding during treatment. No patients received anticoagulant therapy.

Overall, following the aforementioned therapies, eight patients improved, while unfortunately, one patient died. Among the eight patients who exhibited a positive response to the treatment, four patients exhibited improvement in the oxygenation index as determined by blood gas analysis within 48–72 h of therapy, while the remaining four underwent lung CT re-evaluation after two weeks of treatment, revealing lesion absorption. Among the four patients with cerebral involvement, three comatose individuals regained consciousness following a 10-day course of supportive therapy and corticosteroid treatment. The remaining awake patient underwent a brain CT re-examination after two weeks of supportive treatment, revealing significant absorption of the lesion. In summary, all eight patients received respiratory support, with six of them additionally undergoing corticosteroid therapy. Among four patients with cerebral involvement, three demonstrated significant symptom resolution following a 2-week treatment, as evidenced by CT scans showing lesion absorption. Additionally, they had no residual cerebral infarction sequelae. Patient 3 developed an embolism in the left carotid artery, resulting in a cerebral infarction that affected the temporal, parietal and occipital lobes. This ultimately led to a comatose state. Upon admission to the ICU, mechanical ventilation and corticosteroid treatment were administered; however, brain death ultimately occurred (Table 2).

## Discussion

Fractures, especially long bone and pelvis fractures, are the most common cause of FES. Historically, the incidence of FES caused by fractures has been reported to be as high as 30%; however, recent studies have indicated a gradual decrease to 1.69% over time [10, 11]. There are other risk factors for FES, including pancreatitis, bone tumour lysis, sickle cell disease and notably liposuction. Liposuction is one of the most popular body contouring surgeries worldwide. However, with increased surgeries, its life-threatening complications, such as FES, have drawn increasing attention [12]. This is a comprehensive analysis of the characteristics of patients diagnosed with FES caused by fracture or liposuction, which has provided valuable insights into clinical manifestations and disease progression. This information can aid clinicians in making early diagnoses and determining the optimal treatment timing.

El-Ali and Gourlay [4] reported an animal study that demonstrated systemic fat mobilization and fat embolism after liposuction. In his study, 10 rats underwent liposuction on the groin area. All animals had evidence of pulmonary fat embolism, while one had evidence of cerebral fat embolism. Patients 1, 2 and 4 who underwent thigh liposuction all exhibited pulmonary fat embolism, which was consistent with the conclusion of a previous study that liposuction is a risk factor for pulmonary fat embolism. In addition, patients who undergo autologous facial fat grafting are more likely to develop cerebral fat embolism [13]. The fat globules that enter the terminal branches of the ophthalmic artery can reach the internal carotid artery due to both the facial arterial network and injection pressure. Interestingly, patient 5 developed pulmonary fat embolism following facial fat grafting, which is rare. We suspected that this was due to the fat being injected into the veins of the face. The face contains various venous networks, and as such, the fat globules injected into these veins can drain into the vena jugularis interna and ultimately reach the pulmonary artery. Due to the pressure exerted during injection, patients may experience pulmonary symptoms within a short time. Previous studies have reported two cases of pulmonary fat embolism after facial fat grafting [14, 15]. Moreover, the onset of symptoms in patients with orthopaedic fractures was relatively delayed, typically manifesting at approximately 24 h postinjury, which is notably later than that in patients undergoing liposuction, where symptoms appear at approximately 1.8 h. This phenomenon can be explained by the biochemical theory. Bone marrow fat embolism in the local veins leads to the release of lipase, which breaks down into glycerol and free fatty acids. Toxic products damage endothelial cells and trigger an inflammatory response, which may result in acute respiratory distress syndrome [16].

Laboratory examinations upon admission to the ICU revealed that six patients presented with increased white blood cells or reduced haemoglobin and four patients exhibited decreased platelet counts, which were consistent with findings in FES patients [8]. However, these findings could also be attributed to inflammation, bleeding or fluid therapy induced by liposuction or orthopaedic injuries [17]. The CT findings of pulmonary FES commonly include ground-glass opacities, patchy exudation and small nodules of various sizes. A previous study demonstrated that the extent of patchy exudation and ground-glass opacities is positively correlated with disease severity. In our case, although patient 1’s initial lung CT scan revealed diffuse lesions in both lungs, a follow-up examination within 48–72 h showed significant improvement in lung absorption. Furthermore, CT re-examination of three patients with cerebral infarction two weeks after treatment revealed improved lesions compared to their previous state. These findings indicate that the observed change is reversible, and prompt and effective treatment is crucial for significantly improving both survival rates and prognosis [12].

Supportive care for maintaining ventilation and haemodynamics is critical. All patients received respiratory support, and ICU admission was necessary for further therapy for all patients. Corticosteroids have been proposed as treatments for FES due to their ability to suppress the inflammatory cascade and immune response resulting from biochemical damage caused by fat embolism [8, 18] but these effects remain controversial. In our study, all nine patients developed lung or diffuse lung imaging changes. The use of corticosteroids may reduce pulmonary oedema, decrease pulmonary vascular leakage and improve pulmonary ventilation. Therefore, for patients with diffuse lesions in both lungs and hypoxemia, early initiation of glucocorticoid therapy combined with respiratory support could improve patient prognosis. However, the specific dosage, treatment duration and adverse effects remain undefined and require further investigation. Additionally, the timing of surgical intervention is important for patients with orthopaedic fractures. Stinner and Edwards [19] reported that early stabilization of fractures can reduce the risk of extensive FES and shorten hospital stays. Therefore, clinicians should perform comprehensive preoperative evaluations and determine the optimal timing of surgery.

Our study has several limitations. First, the major limitation is that the number of patients included was not large, which restricted further exploration of disease manifestations. Second, as a retrospective study, we were unable to obtain complete medical data for some patients. Despite these limitations, we conducted a systematic analysis of clinical and radiological characteristics.

## Conclusions

FES is a rare but fatal complication that can occur in patients with long bone fractures or who are undergoing liposuction. Due to the non-specificity of clinical manifestations, early diagnosis can be challenging. Clinicians should pay close attention to medical history and consider timely diagnosis of FES if patients present with respiratory, neurological or cardiovascular symptoms. Early clinical suspicion, diagnosis and timely treatment are associated with a favourable prognosis.

## Data Availability

The data that support the findings of this study are available from the corresponding author, JLK, upon reasonable request.
